# Perspectives of Oncologists on the Ethical Implications of Using Artificial Intelligence for Cancer Care

**DOI:** 10.1001/jamanetworkopen.2024.4077

**Published:** 2024-03-28

**Authors:** Andrew Hantel, Thomas P. Walsh, Jonathan M. Marron, Kenneth L. Kehl, Richard Sharp, Eliezer Van Allen, Gregory A. Abel

**Affiliations:** 1 Divsion of Population Sciences, Dana-Farber Cancer Institute, Boston, Massachusetts; 2 Harvard Medical School, Boston, Massachusetts; 3 Harvard Medical School Center for Bioethics, Boston, Massachusetts; 4 Divsion of Pediatric Hematology/Oncology, Boston Children’s Hospital, Boston, Massachusetts; 5 Divsion of Health Care Policy & Research, Mayo Clinic, Rochester, Minnesota; 6 Broad Institute, Cambridge, Massachusetts

## Abstract

**Importance:**

Artificial intelligence (AI) tools are rapidly integrating into cancer care. Understanding stakeholder views on ethical issues associated with the implementation of AI in oncology is critical to optimal deployment.

**Objective:**

To evaluate oncologists’ views on the ethical domains of the use of AI in clinical care, including familiarity, predictions, explainability (the ability to explain how a result was determined), bias, deference, and responsibilities.

**Design, Setting, and Participants:**

This cross-sectional, population-based survey study was conducted from November 15, 2022, to July 31, 2023, among 204 US-based oncologists identified using the National Plan & Provider Enumeration System.

**Main Outcomes and Measures:**

The primary outcome was response to a question asking whether participants agreed or disagreed that patients need to provide informed consent for AI model use during cancer treatment decisions.

**Results:**

Of 387 surveys, 204 were completed (response rate, 52.7%). Participants represented 37 states, 120 (63.7%) identified as male, 128 (62.7%) as non-Hispanic White, and 60 (29.4%) were from academic practices; 95 (46.6%) had received some education on AI use in health care, and 45.3% (92 of 203) reported familiarity with clinical decision models. Most participants (84.8% [173 of 204]) reported that AI-based clinical decision models needed to be explainable by oncologists to be used in the clinic; 23.0% (47 of 204) stated they also needed to be explainable by patients. Patient consent for AI model use during treatment decisions was supported by 81.4% of participants (166 of 204). When presented with a scenario in which an AI decision model selected a different treatment regimen than the oncologist planned to recommend, the most common response was to present both options and let the patient decide (36.8% [75 of 204]); respondents from academic settings were more likely than those from other settings to let the patient decide (OR, 2.56; 95% CI, 1.19-5.51). Most respondents (90.7% [185 of 204]) reported that AI developers were responsible for the medico-legal problems associated with AI use. Some agreed that this responsibility was shared by physicians (47.1% [96 of 204]) or hospitals (43.1% [88 of 204]). Finally, most respondents (76.5% [156 of 204]) agreed that oncologists should protect patients from biased AI tools, but only 27.9% (57 of 204) were confident in their ability to identify poorly representative AI models.

**Conclusions and Relevance:**

In this cross-sectional survey study, few oncologists reported that patients needed to understand AI models, but most agreed that patients should consent to their use, and many tasked patients with choosing between physician- and AI-recommended treatment regimens. These findings suggest that the implementation of AI in oncology must include rigorous assessments of its effect on care decisions as well as decisional responsibility when problems related to AI use arise.

## Key Points

**Question:**

What are oncologists’ views on ethical issues associated with the implementation of artificial intelligence (AI) in cancer care?

**Findings:**

In this cross-sectional survey study, 84.8% of US oncologists reported that AI needs to be explainable by oncologists but not necessarily patients, and 81.4% agreed that patients should consent to AI use for cancer treatment decisions. Less than half (47.1%) of oncologists viewed medico-legal problems from AI use as physicians’ responsibility, and although most (76.5%) reported feeling responsible for protecting patients from biased AI, few (27.9%) reported feeling confident in their ability to do so.

**Meaning:**

This study suggests that concerns about ethical issues, including explainability, patient consent, and responsibility, may impede optimal adoption of AI into cancer care.

## Teaser

This cross-sectional survey study evaluates oncologists’ views on the ethical domains of the use of artificial intelligence (AI) in clinical care, including familiarity, predictions, explainability, bias, deference, and responsibilities.

## Introduction

Artificial intelligence (AI) is an emerging set of technologies with the potential to advance cancer discovery and care delivery.^1^ Artificial intelligence models with applications for oncology have recently been approved by the US Food and Drug Administration (FDA),^2^ and the increasing complexity of personalized cancer care makes the field of oncology poised for an AI revolution. Concerns have been raised over AI bias, explainability (ie, the ability of an AI model to explain how it reached a result), responsibility for error or misuse, and humans’ deference to its results.^3,4,5^ As the ethical deployment of AI in cancer care requires solutions that meet the needs of stakeholders, this study sought to examine oncologists’ familiarity with AI and perspectives on these issues. As familiarity with a technology changes stakeholder perceptions of it,^6^ and because academic research in AI is burgeoning, we hypothesized that responses would vary for oncologists practicing in academic settings compared with those in other practice settings.

## Methods

From November 15, 2022, to July 31, 2023, we performed a cross-sectional survey study of oncologists practicing in the US. A draft instrument based on published ethical frameworks^4,5^ was developed by a team of oncologists, survey methodologists, bioethicists, and AI researchers (A.H., T.P.W., J.M.M., K.L.K., R.S., E.V.A., and G.A.A.). The instrument was iteratively refined through cognitive testing with 5 practicing oncologists until meaning saturation was achieved. The final instrument (eMethods in Supplement 1) contained 24 questions including demographics and the following domains: AI familiarity, predictions, explainability, bias, deference, and responsibilities. A random sample of oncologists was identified using the National Plan & Provider Enumeration System (eMethods in Supplement 1).^7^ Recruitment methods followed best practices,^8^ using mailed paper surveys with gift cards ($25), after which reminder letters with an electronic survey option and telephone calls were used for nonresponders. The study was approved by the Dana-Farber Office for Human Research Studies. We received a waiver of written documentation of consent from the Dana-Farber Cancer Institute institutional review board. The survey instrument was introduced with a clear consent statement (a full page on paper and a full screen in the electronic version) describing the study, its voluntary nature, the participant’s rights, and what participation entailed. Completing the survey constituted consent to participate in the study. This study followed the CROSS guidelines^9^ (eMethods in Supplement 1).

Responses were grouped for analysis as shown in the eMethods in Supplement 1. The χ^2^ test or the Fisher exact test assessed bivariate associations between responses and primary practice (academic hospital or clinic [“academic”] vs other), with odds ratios (ORs) and 95% CIs reported. The primary outcome was respondent views on the need for patients to provide informed consent for the use of an AI model during treatment decision-making. A multivariable logistic regression model assessed associations between respondent characteristics with the primary outcome; covariates with *P* ≤ .05 in bivariate testing were included. These covariates included sociodemographic characteristics (including self-reported race and ethnicity [racial and ethnic group categories were aligned with National Institutes of Health reporting guidelines under NOT-OD-15-089; race and ethnicity were assessed because a number of AI tools have been shown to perpetuate bias and racism that inordinately affects minoritized racial and ethnic groups]), practice setting, and prior training, defined as previous AI-specific education (eg, courses and lectures). Imputation was planned if question missingness was more than 5%. All *P* values were 2-sided; the significance level was *P* < .05 unless otherwise specified. Statistical analyses were performed using Stata, version 16 (StataCorp LLC).

## Results

Of 399 mailed surveys, 12 were undeliverable, and 204 were completed (response rate, 52.7%); question missingness was less than 1%. Participants represented 37 states, 120 (63.7%) identified as male, 128 (62.7%) identified as non-Hispanic White, and 60 (29.4%) were from academic practices; 109 (53.4%) had no prior AI training, and 45.3% (92 of 203) reported familiarity with clinical decision models (Table 1). Although 93.1% (189 of 203) reported that they would benefit from dedicated training, 75.0% (153 of 204) did not know of appropriate resources. eTables 1 to 4 in Supplement 1 show familiarity, predictions, and acceptability of AI models. Those in academic practices were more likely than those in other settings to report they could explain AI pathology models (OR, 2.08; 95% CI, 1.06-4.12). They were also more likely to predict that AI would improve adverse effect management (OR, 1.93; 95% CI, 1.01-3.73) and end-of-life decision-making (OR, 2.06; 95% CI, 1.11-3.84).

**Table 1.** Self-Reported Respondent Characteristics

Characteristic	Respondents, No. (%)			*P* value^a^
	All (N = 204)	Practice setting (n = 202)		
		Academic (n = 60)	Other (n = 142)	
Age group, y				
<40	45 (22.1)	18 (30.0)	27 (19.0)	.12
40-59	112 (54.9)	30 (50.0)	81 (57.0)	
60-80	46 (22.5)	11 (18.3)	34 (23.9)	
>80	1 (0.5)	1 (1.7)	0	
Gender				
Female	72 (35.3)	20 (33.3)	51 (35.9)	.68
Male	130 (63.7)	40 (66.7)	89 (62.7)	
Unknown	2 (1.0)	0	0	
Race and ethnicity				
Asian Indian	34 (16.7)	6 (10.0)	28 (19.7)	.36
Black or African American	9 (4.4)	4 (6.7)	5 (3.5)	
Eastern Asian or Other Pacific Islander	20 (9.8)	5 (8.3)	14 (9.9)	
White	128 (62.7)	42 (70.0)	84 (59.2)	
Other^b^	10 (4.9)	2 (3.3)	8 (5.6)	
≥1 Race	3 (1.5)	0	3 (2.1)	
Hispanic origin				
Yes	12 (5.9)	4 (6.7)	8 (5.6)	.78
No	192 (94.1)	56 (93.3)	134 (94.4)	
Years in practice				
≤5	33 (16.2)	13 (21.7)	20 (14.1)	.58
6-10	31 (15.2)	10 (16.7)	21 (14.8)	
11-20	74 (36.3)	20 (33.3)	53 (37.3)	
21-30	41 (20.1)	12 (20.0)	28 (19.7)	
≥31	25 (12.3)	5 (8.3)	20 (14.1)	
Oncology specialty				
Medical oncology	126 (61.8)	32 (53.3)	92 (64.8)	.16
Radiation oncology	56 (27.5)	18 (30.0)	38 (26.8)	
Surgical oncology	22 (10.8)	10 (16.7)	12 (8.5)	
Familiar with ≥2 AI model types				
Yes	141 (69.1)	44 (73.3)	96 (67.6)	.45
No	62 (30.4)	15 (25.0)	46 (32.4)	
Unknown	1 (0.5)	1 (1.7)	0	
Prior AI training				
Yes	95 (46.6)	44 (73.3)	50 (35.2)	<.001
No	109 (53.4)	16 (26.7)	92 (64.8)	
Practice setting				
Academic	60 (29.4)	NA	NA	NA
Other	142 (69.6)	NA	NA	NA
Unknown	2 (1.0)	NA	NA	NA

Abbreviations: AI, artificial intelligence; NA, not applicable.

^a^ Determined by the χ^2^ or Fisher exact test.

^b^ Other race or ethnicity was a free-text response on the survey and was not an aggregated response of predefined categories.

**Figure 1.**
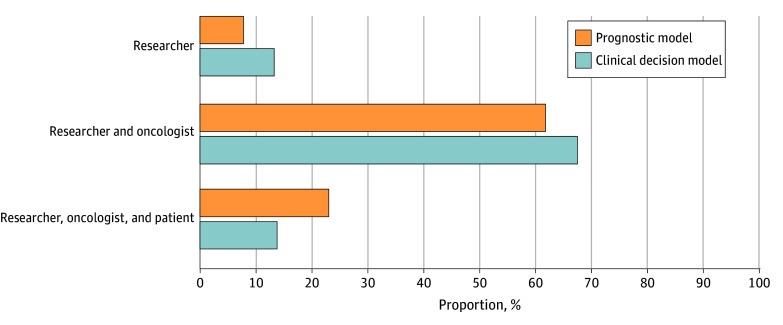
Responses to 2 Questions Assessing Which Stakeholder Types (Researcher, Oncologist, or Patient) Should Be Able to Explain an Artificial Intelligence Model for It to Be Used in Clinic Responses of “none” or “never” constituted less than 5% of the sample and are not shown.

**Figure 2.**
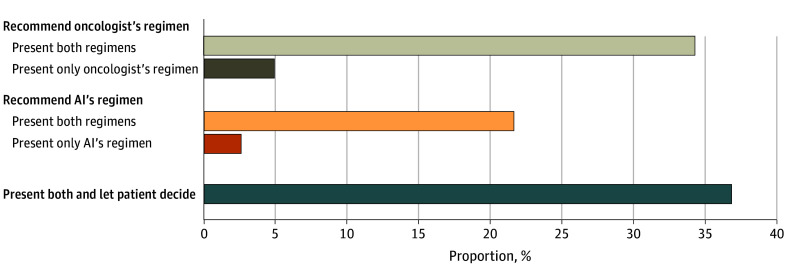
Responses to a Scenario Where a US Food and Drug Administration–Approved Artificial Intelligence (AI) Model Selects a Different Regimen Than the Oncologist Planned to Recommend

Few participants reported that AI prognostic (13.2% [27 of 203]) and clinical decision (7.8% [16 of 204]) models could be used clinically when only researchers could explain them; 81.3% (165 of 203) and 84.8% (173 of 204), respectively, reported they needed to be explainable by oncologists, while 13.8% (28 of 203) and 23.0% (47 of 204), respectively, stated they also needed to be explainable by patients (Figure 1). Those from academic practices were less likely than those from other practices to view patient explainability as necessary (OR, 0.25; 95% CI, 0.10-0.64). When presented with a scenario in which an FDA-approved AI decision model selected a different regimen than the oncologist initially planned to recommend (eMethods in Supplement 1; Figure 2), the most common response was to present both options and let the patient decide (36.8% [75 of 204]); this proportion was consistent in a subanalysis limited to those who reported that decision models did not need to be explainable by patients (34.5% [51 of 148]). Differences by grouped responses (oncologist’s recommendation, AI’s recommendation, or patient’s decision; Figure 2) were seen by practice setting (χ^2^ = 9.35; *P* = .009). In pairwise comparisons (threshold of significance, Bonferroni-corrected *P* < .017), respondents from academic practices were more likely than those from other practices to choose the AI’s recommendation over their initial recommendation (OR, 2.99; 95% CI, 1.39-6.47; Bonferroni-corrected *P* = .004) or defer the decision to the patient (OR, 2.56; 95% CI, 1.19-5.51; Bonferroni-corrected *P* = .02).

More respondents reported that patients should consent to the use of AI tools in treatment decisions (81.4% [166 of 204]) than diagnostic decisions (56.4% [115 of 204]). Bivariate associations were seen between supporting consent for AI use during treatment decisions and not practicing in an academic setting (compared with an academic setting; OR, 2.39; 95% CI, 1.13-5.06) as well as not having prior AI training (compared with having prior training; OR, 2.81; 95% CI, 1.32-6.00); other associations were not significant (eTable 5 in Supplement 1). In a multivariable model, the association between preference for consent and lack of prior AI training was retained (OR, 2.62; 95% CI, 1.15-5.95), but practice setting was not (OR, 1.71; 95% CI, 0.77-3.82) (Table 2).

**Table 2.** Multivariable Logistic Regression Model of Preference for Patient Consent to the Use of a Treatment Decision AI Model by Demographic Characteristics^a^

Characteristic	Odds ratio (95% CI)	*P* value
Practice setting		
Primary academic	1 [Reference]	.19
Other	1.72 (0.77-3.82)	
Prior AI training		
Yes	1 [Reference]	.02
No	2.62 (1.15-5.96)	

Abbreviation: AI, artificial intelligence.

^a^ Only characteristics with significant bivariate associations were retained.

Most respondents (90.7% [185 of 204]) reported that AI developers should be responsible for the medico-legal problems associated with AI. Fewer reported that responsibility was shared by physicians (47.1% [96 of 204]) and/or hospitals (43.1% [88 of 204]). Most respondents (76.5% [156 of 204]) agreed that oncologists should protect patients from biased AI. Only 27.9% (57 of 204) of respondents were confident in their ability to identify how representative the data used in an AI model were, including 66.0% (103 of 156) of those who reported it was the oncologists’ responsibility to protect patients from biased tools. Respondents from academic practices were more likely to report confidence identifying representative AI (OR, 2.73; 95% CI, 1.43-5.23) and were as likely as respondents from other practices to report a responsibility to protect patients from biased tools (OR, 0.99; 95% CI, 0.49-2.03).

## Discussion

In this nationally representative, cross-sectional survey study assessing oncologists’ views on ethical issues associated with AI in cancer care, we found associations between practice setting and AI-related predictions, deference, and explainability. Most participants reported that patients should consent to the use of AI during treatment decision-making, and those without prior training were more likely to view consent as necessary. Responses about decision-making were sometimes paradoxical; patients were not expected to understand AI tools but were expected make decisions related to recommendations generated by AI. A gap was also seen between oncologist responsibilities and preparedness to combat AI-related bias. Together, these data characterize barriers that may impede the ethical adoption of AI into cancer care.

There is relatively little known about AI’s clinical implementation issues as they relate to clinical stakeholders.^10^ Our findings begin to bridge AI development with the expectations of end users so that tools can be appropriately applied. For example, oncologists’ knowledge and training were relatively uncommon compared with self-reported obligations to patients and deference to AI. This finding complements normative discussions about the erosion of human responsibilities through AI overreliance^11^ and brings up the question about whether such responsibilities will always be necessary. This aligns with our finding that few respondents assumed responsibility for the medico-legal problems stemming from AI recommendations.

### Limitations

This study has some limitations, including the moderate sample size and response rate, although cohort demographics appear to be nationally representative.^12,13^ In addition, responses to specific use cases and thresholds for using AI may differ from the general perceptions identified. Psychometrically validated AI-focused survey instruments were not available, but pretesting was used to enhance face and content validity. Finally, the cross-sectional nature of these data limits generalizability over time as AI is integrated into cancer care.

## Conclusions

Ethical AI in cancer care requires accounting for stakeholder positions. This cross-sectional survey study highlights potential issues related to accountability and deference to AI as well as associations with practice setting. Our findings suggest that the implementation of AI in the field of oncology must include rigorous assessments of its effect on care decisions and decisional responsibility when problems related to AI use arise.

## Notes

Supplement 1.
eMethods.
**eTable 1.** Respondent Familiarity and Ability to Explain AI Model Types (N=203) **eTable 2.** Respondent General Predictions Related to AI (N=203) **eTable 3.** Respondent Clinical Predictions Related to AI (N=203) **eTable 4.** Respondent Views on Acceptability of Direct-to-Patient AI Model Applications (N=204) **eTable 5.** Bivariate Logistic Regressions Between Demographic Characteristics and Preference for Patient Consent to the Use of a Treatment Decision AI Model
eReference.

Supplement 2.
Data Sharing Statement
