# Supplementary material for: Perspectives of Oncologists on the Ethical Implications of Using Artificial Intelligence for Cancer Care
Source: JAMA Netw Open. 2024 Mar 28;7(3):e244077. doi: 10.1001/jamanetworkopen.2024.4077 (PMC10979310; doi:10.1001/jamanetworkopen.2024.4077)
Supplement: Supplement 1. — eMethods. eTable 1. Respondent Familiarity and Ability to Explain AI Model Types (N=203) eTable 2. Respondent General Predictions Related to AI (N=203) eTable 3. Respondent Clinical Predictions Related to AI (N=203) eTable 4. Respondent Views on Acceptability of Direct-to-Patient AI Model Applications (N=204) eTable 5. Bivariate Logistic Regressions Between Demographic Characteristics and Preference for Patient Consent to the Use of a Treatment Decision AI Model eReference. [file jamanetwopen-e244077-s001.pdf]

# Supplementary Online Content

Hantel A, Walsh TP, Marron JM, et al. Perspectives of oncologists on the ethical implications of using artificial intelligence for cancer care. *JAMA Netw Open.* 2024;7(3):e244077. doi:10.1001/jamanetworkopen.2024.4077

**eMethods.**

**eTable 1.** Respondent Familiarity and Ability to Explain AI Model Types (N=203)

**eTable 2.** Respondent General Predictions Related to AI (N=203)

**eTable 3.** Respondent Clinical Predictions Related to AI (N=203)

**eTable 4.** Respondent Views on Acceptability of Direct-to-Patient AI Model Applications (N=204)

**eTable 5.** Bivariate Logistic Regressions Between Demographic Characteristics and Preference for Patient Consent to the Use of a Treatment Decision AI Model

**eReference.**

This supplementary material has been provided by the authors to give readers additional information about their work.

© 2024 Hantel A et al. *JAMA Network Open.*

**eMethods.**

**National Plan & Provider Enumeration System Sampling Methods**

The "Full Replacement Monthly NPI File" was downloaded from https://www.cms.gov/Regulations-and-Guidance/Administrative-Simplification/NationalProvIdentStand/DataDissemination in December 2022. The following filtering criteria were applied to the dataset to minimize out-of-date information, individuals and entities that did not match the study's intended respondents:

| Variable | Filter |
|---|---|
| Provider Taxonomy Codes | 207RH0003X (Allopathic & Osteopathic Physicians/Internal Medicine Hematology & Oncology)<br>207RX0202X (Allopathic & Osteopathic Physicians/Internal Medicine Medical Oncology)<br>207VX0201X (Allopathic & Osteopathic Physicians/Obstetrics & Gynecology Gynecologic Oncology)<br>2080P0207X (Allopathic & Osteopathic Physicians/Pediatrics Pediatric Hematology-Oncology)<br>2085R0001X (Allopathic & Osteopathic Physicians/Radiology Radiation Oncology)<br>2086X0206X (Allopathic & Osteopathic Physicians/Surgery Surgical Oncology) |
| Organization Name | Excluded organization NPIs |
| Provider Credential Text | Retained individuals with "MD," "DO," and "MBBS" degrees; individuals with degrees in addition to these were also retained (e.g., "MD, PhD") |
| Provider First Line Practice Mailing Address | Excluded PO Box Mailing Addresses |
| Provider Practice Mailing Address Country | Retained "United States" or "US" |
| Provider Telephone Number | Excluded those without telephone numbers |
| Last Update Year | 2018-2022 |
| Certification Date | Excluded those without certification |

A randomizing function was then performed among retained NPI listings. Two study team members verify the mailing address and telephone number listed through internet searches and telephone calls, those with unverifiable address and/or telephone numbers were excluded. 500 individuals were screened for contact information; 101 were excluded for unverifiable information and 399 were retained and constituted the final study sample.

© 2024 Hantel A et al. *JAMA Network Open.*

**Final Survey Instrument**

No._______

Dana-Farber Cancer Institute

# The Oncologist's Perspective on Artificial Intelligence

We are seeking to better understand oncologists' views on artificial intelligence in cancer care. Your participation is valuable and will help us develop services for oncologists as artificial intelligence tools are implemented for cancer care.

- This **one-time survey** will take approximately **10 minutes** to complete.
- Your participation is voluntary, and your answers will be kept confidential. **You will only be identified by the number on your survey.**
- You may opt out of the study by returning the postcard included in this packet or leaving a voicemail for the study staff at (617) 582-9394.
- If you come across a question you would rather not answer, it is okay to skip it and go on to the next question.
- If you have any questions please contact the principal investigator, Dr. Andrew Hantel, at (617) 582-9394 or by email at andrew_hantel@dfci.harvard.edu.

**When you have completed the survey, please place it in the pre-stamped and addressed envelope provided and mail it back.**

**Intentionally left blank**

## About You

The following questions ask about your background. Please answer to the best of your ability. If you prefer not to answer a question, you may skip to the next question.

1. What is your age in years?
*Select only one.*
☐ Less than 40 years old
☐ 40-59 years old
☐ 60-80 years old
☐ 81 or more years old

2. What is your gender?
*Select only one.*
☐ Female
☐ Male
☐ I do not identify as female or male

3. Are you Hispanic, Latino/a/x, or of Spanish origin?
*Select only one.*
☐ No, I am not of Hispanic, Latino/a/x, or Spanish origin
☐ Yes, I am of Hispanic, Latino/a/x or Spanish origin (for example: Mexican, Chicano/a, Puerto Rican)

4. What is your race?
*Select one or more.*
☐ Asian Indian
☐ American Indian or Alaska Native
☐ Black or African American
☐ Eastern Asian or Pacific Islander (for example: Chamorro, Chinese, Filipino, Japanese, Korean, Samoan, Vietnamese, Native Hawaiian)
☐ White
☐ A race not listed: ________________ *Fill in*

5. What is your primary oncology subspecialty?
*Select only one.*
☐ Medical oncology
☐ Surgical oncology
☐ Radiation oncology
☐ Gynecologic oncology
☐ Neurologic oncology
☐ Pediatric oncology

6. What best describes your primary practice location?
*Select only one.*
☐ Primary academic hospital or clinic
☐ Other hospital or clinic with an academic affiliation
☐ Community hospital or clinic with no academic affiliation

7. For how many years have you been in practice after the completion of your oncology training?
*Select only one.*
☐ 0-5 years
☐ 6-10 years
☐ 11-20 years
☐ 21-30 years
☐ 31 or more years

CONTINUE TO THE NEXT PAGE

## About Artificial Intelligence

The following items ask about your views on various questions and statements surrounding the use of artificial intelligence in oncology.

For the purposes of this survey, **artificial intelligence (AI) is defined as: "the ability of a machine to learn and recognize patterns and interactions from representative data, and to use this information for improving the process of decision-making."**

8. I am familiar with the following applications of artificial intelligence in cancer care.
*Select one response per row.*

| **Application** (Explanation of an FDA-approved example) | Strongly Disagree | Disagree | Agree | Strongly Agree |
|---|---|---|---|---|
| **Pathology Models** (An AI model that interprets biopsies for cancer diagnosis) | ☐ | ☐ | ☐ | ☐ |
| **Radiology Models** (An AI model that interprets radiological images for cancer diagnosis) | ☐ | ☐ | ☐ | ☐ |
| **Prognostic Models** (An AI model that predicts clinical endpoints including overall survival) | ☐ | ☐ | ☐ | ☐ |
| **Clinical Decision Models** (An AI model that provides diagnostic and treatment decision support) | ☐ | ☐ | ☐ | ☐ |

9. I can explain the results of the following types of artificial intelligence models to my patients.
*Select one response per row.*

| **Application** | Strongly Disagree | Disagree | Agree | Strongly Agree |
|---|---|---|---|---|
| **Pathology Models** | ☐ | ☐ | ☐ | ☐ |
| **Radiology Models** | ☐ | ☐ | ☐ | ☐ |
| **Prognostic Models** | ☐ | ☐ | ☐ | ☐ |
| **Clinical Decision Models** | ☐ | ☐ | ☐ | ☐ |

© 2024 Hantel A et al. *JAMA Network Open.*

**10.** I am familiar with the following method(s) of statistical prediction.
*Select one or more.*

- ☐ Multivariable regression model
- ☐ Neural network
- ☐ Bayesian method
- ☐ Random forest
- ☐ Cox regression model
- ☐ Decision tree
- ☐ k-nearest neighbor
- ☐ Gradient boosted machines

- ☐ None of these

**11.** In the next 10 years, artificial intelligence will improve …
*Select one response per row.*

| | Strongly Disagree | Disagree | Agree | Strongly Agree |
|---|---|---|---|---|
| … cancer diagnosis. | ☐ | ☐ | ☐ | ☐ |
| … cancer treatment. | ☐ | ☐ | ☐ | ☐ |
| … management of treatment side effects. | ☐ | ☐ | ☐ | ☐ |
| … end-of-life decision-making. | ☐ | ☐ | ☐ | ☐ |

**12.** In the next 10 years, artificial intelligence will …
*Select one response per row.*

| decrease | not change | increase | |
|---|---|---|---|
| ☐ | ☐ | ☐ | … the trust patients place in oncologists. |
| ☐ | ☐ | ☐ | … oncologists' skills. |
| ☐ | ☐ | ☐ | … disparities in cancer care. |

**13.** Artificial intelligence will ________ the cancer care I provide.
*Select only one.*

- ☐ Depersonalize
- ☐ Not change
- ☐ Personalize

**14.** An accurate and unbiased artificial intelligence <u>prognostic</u> model should …
*Select only one.*

- ☐ … be used in clinic as long as <u>researchers</u> can explain how they work.
- ☐ … be used in clinic as long as <u>researchers and oncologists</u> can explain how they work.
- ☐ … be used in clinic as long as <u>researchers, oncologists, and patients</u> can explain how they work.
- ☐ … be used in clinic even if <u>researchers, oncologists, and patients can't</u> explain how they work.
- ☐ … never be used in clinic.

**15.** An accurate and unbiased artificial intelligence <u>clinical decision</u> model should …
*Select only one.*

- ☐ … be used in clinic as long as <u>researchers</u> can explain how they work.
- ☐ … be used in clinic as long as <u>researchers and oncologists</u> can explain how they work.
- ☐ … be used in clinic as long as <u>researchers, oncologists, and patients</u> can explain how they work.
- ☐ … be used in clinic even if <u>researchers, oncologists, and patients can't</u> explain how they work.
- ☐ … never be used in clinic.

**16.** It would be acceptable for oncology patients to interact with the following types of artificial intelligence models directly (i.e., without a healthcare provider) if they are FDA-approved:
*Select one or more.*

- ☐ Natural language processing (e.g., automated translation of provider notes into lay language)
- ☐ Clinical trial matching
- ☐ Supportive care counseling (e.g., nutritional guidance)
- ☐ Medical decision-aides (e.g., at-home skin lesion screening)

**17.** I am confident in my ability to identify how well the dataset an artificial intelligence model was trained on represents the population with that condition.
*Select only one.*

- ☐ Strongly disagree
- ☐ Disagree
- ☐ Agree
- ☐ Strongly agree

**18.** It is oncologists' responsibility to protect their patients from biased artificial intelligence tools.
*Select only one.*

- ☐ Strongly disagree
- ☐ Disagree
- ☐ Agree
- ☐ Strongly agree

**19.** The following entity or entities should be responsible for medico-legal problems caused by using an FDA-approved artificial intelligence model in clinical cancer care:
*Select one or more.*

- ☐ The physician who used the model.
- ☐ The company or organization that developed the model.
- ☐ The hospital or institution under which the care was being provided.

**20.** Patients should provide informed consent for the use of an artificial intelligence model in …
*Select one response per row.*

| | Strongly Disagree | Disagree | Agree | Strongly Agree |
|---|---|---|---|---|
| … clinical cancer diagnosis. | ☐ | ☐ | ☐ | ☐ |
| … cancer treatment decisions. | ☐ | ☐ | ☐ | ☐ |

**21.** You see a patient with previously untreated metastatic colon cancer in clinic. Based on your review of their case, you plan to propose chemotherapy regimen *A*. Before discussing this with the patient, you use an FDA-approved artificial intelligence model to assess their options, and it proposes that the patient receive chemotherapy regimen *B*. Both regimen *A* and regimen *B* are FDA-approved in this setting, fall under the same category of recommendation in a widely used clinical practice guideline, and have similar costs, side effects, and treatment schedules. You would …
*Select only one.*

- ☐ Present and recommend regimen *A* only.
- ☐ Present and recommend regimen *B* only.
- ☐ Present both options and recommend regimen *A*.
- ☐ Present both options and recommend regimen *B*.
- ☐ Present both options and let the patient decide.

**22.** I have received the following types of training on artificial intelligence in cancer care:
*Select one or more.*

- ☐ Lectures
- ☐ Workshops and/or Conferences
- ☐ Formal courses
- ☐ Self-learning
- ☐ Certificate programs
- ☐ Advanced degrees (e.g., Master's, Doctorate)

- ☐ I have not received training on artificial intelligence

**23.** I know where to find resources to teach myself about artificial intelligence for cancer care.
*Select only one.*

- ☐ Strongly disagree
- ☐ Disagree
- ☐ Agree
- ☐ Strongly agree

**24.** I would benefit from dedicated training on artificial intelligence for cancer care.
*Select only one.*

- ☐ Strongly disagree
- ☐ Disagree
- ☐ Agree
- ☐ Strongly agree

**Thank you for completing the survey!**

**Please place your completed survey in the pre-stamped and addressed envelope provided and mail it back.**

No.________

© 2024 Hantel A et al. *JAMA Network Open.*

**Question Response Groupings for Analysis**

If questions are not listed, responses were analyzed as shown in the survey.

| Question(s) | Question/Response Category | Grouping for Analysis | Notes |
|---|---|---|---|
| 3, 4 | Asian Indian | People of Color | Asian Indian, American Indian or Alaska Native, Black or African American, Eastern Asian or Pacific Islander were aggregated as People of Color independent of response to question 3. |
| | American Indian or Alaska Native | People of Color | |
| | Black or African American | People of Color | |
| | Eastern Asian or Pacific Islander | People of Color | |
| | White AND Not of Hispanic, Latino/a/x, or Spanish Origin | Non-Hispanic White | |
| | White AND Hispanic, Latino/a/x, or Spanish Origin | People of Color | |
| 5 | Medical oncology | Medical Oncology | |
| | Surgical oncology | Surgical Oncology | |
| | Radiation oncology | Radiation Oncology | |
| | Gynecologic oncology | Surgical Oncology | |
| | Neurologic oncology | Medical Oncology | |
| | Pediatric oncology | Medical Oncology | |
| 6 | Primary academic hospital or clinic | Primary Academic | |
| | Other hospital or clinic with an academic affiliation | Not Primary Academic | |
| | Community hospital or clinic with no academic affiliation | Not Primary Academic | |
| 8, 9, 11, 17, 18, 23, 24 | Strongly Disagree | Disagree | |
| | Disagree | Disagree | |
| | Agree | Agree | |
| | Strongly Agree | Agree | |
| 8 | N/A | Familiar | Familiar if Agree or Strongly Agree for 2 or more model types |
| | | Not Familiar | |
| 9 | N/A | Can explain | Can explain if Agree or Strongly Agree for 2 or more model types |
| | | Can't explain | |
| 10 | Multivariable regression model | Standard Statistical Prediction | |
| | Neural network | AI Statistical Prediction | |
| | Bayesian method | Standard Statistical Prediction | |
| | Random forest | AI Statistical Prediction | |
| | Cox regression model | Standard Statistical Prediction | |
| | Decision tree | Standard Statistical Prediction | |
| | k-nearest neighbor | AI Statistical Prediction | |
| | Gradient boosted machines | AI Statistical Prediction | |
| | None of these | None | |
| 14, 15 | … be used in clinic as long as researchers can explain how they work. | Not explainable by patients | |
| | … be used in clinic as long as researchers and oncologists can explain how they work. | Not explainable by patients | |
| | … be used in clinic as long as researchers, oncologists, and | Explainable by patients | |

© 2024 Hantel A et al. *JAMA Network Open.*

| | | | |
|---|---|---|---|
| | patients can explain how they work. | | |
| | … be used in clinic even if researchers, oncologists, and patients can't explain how they work. | Not explainable by patients | |
| | … never be used in clinic. | Not used | |
| 21 | Present and recommend regimen A only. | Recommend their choice | |
| | Present and recommend regimen B only. | Recommend AI's choice | |
| | Present both options and recommend regimen A. | Recommend their choice | |
| | Present both options and recommend regimen B. | Recommend AI's choice | |
| | Present both options and let the patient decide. | Recommend patient's choice | |
| 22 | Lectures | Prior training | |
| | Workshops and/or Conferences | Prior training | |
| | Formal courses | Prior training | |
| | Self-learning | Prior training | |
| | Certificate programs | Prior training | |
| | Advanced degrees (e.g., Master's, Doctorate) | Prior training | |
| | I have not received training on artificial intelligence | No prior training | |

© 2024 Hantel A et al. *JAMA Network Open.*

**Question and Response Domains and Constructs**

| Question(s) | Domain(s) | Question and Response Construct/Basis |
|---|---|---|
| 8 | Familiarity | Question construct based on general familiarity survey instrument questions. Responses based on categories of AI models approved in cancer care per manuscript reference 2. |
| 9 | Familiarity, Explainability | Question construct based on manuscript reference 5, where explainability is defined as "the doctor must be capable of interpreting the basis on which a result was reached." |
| 10 | Familiarity | Responses based on common types of statistical prediction used in artificial intelligence including from manuscript reference 5 and several reviews including doi: 10.1038/s41746-022-00712-8 and 10.1056/NEJMra2212850 |
| 11 | Prediction | Question construct based on categories of clinical decisions for practicing oncologists. |
| 12, 13 | Prediction | Question construct based on common concerns raised over the interactions of human and AI tools per manuscript references 1, 4, and 5. |
| 14, 15 | Explainability | Question construct based on explainability as defined above (see Q9) and stakeholders as defined in reference 4. Accuracy was held constant to assess explainability thresholds. |
| 16 | Familiarity, Acceptability, | Question construct based on general acceptability survey scales (e.g., the Acceptability of Intervention Measure). Responses based on AI models approved or submitted to the FDA per manuscript reference 2. |
| 17, 18, 19 | Bias, Responsibilitiy | Question construct based on common concerns raised over AI bias per manuscript references 1, 4, and 5 and responsibility per reference 12. |
| 20 | Explainability, Responsibility | Question construct based on explainability as defined above (see Q9) and stakeholders as defined in reference 4 and responsibilities, per manuscript reference 12. |
| 21 | Deference, Responsibility | Question construct based on deference and responsibility as per manuscript references 4, 5, and 12. |
| 22, 23, 24 | Familiarity | Question constructs based on general education and experience instruments. |

© 2024 Hantel A et al. *JAMA Network Open.*

**Additional Survey Methods**

Surveys were sent with cover letters that notified participants of the survey's confidentiality; respondents were only identified on the survey and return mailings through a Study ID that was maintained securely by the study team. Surveys were sent in batches (199 and 200 each) with two opt-out options. Two weeks after the survey was sent a mailed reminder letter was sent to non-respondents. Two weeks after this any non-respondents were called. If they did not respond within two weeks after the phone call, they were considered a non-response. Paper survey data were entered by one team member and these data were verified by a second team member. The electronic survey used a generic link and required participants to enter their Study ID to minimize double entries.

© 2024 Hantel A et al. *JAMA Network Open.*

**Consensus-Based Checklist for Reporting of Survey Studies (CROSS) Reporting Guideline**[1]

| Section/topic | Item | Item description | Reported on page # |
|---|---|---|---|
| **Title and abstract** | | | |
| **Title and abstract** | 1a | State the word "survey" along with a commonly used term in title or abstract to introduce the study's design. | 1 |
| | 1b | Provide an informative summary in the abstract, covering background, objectives, methods, findings/results, interpretation/discussion, and conclusions. | 4-5 |
| **Introduction** | | | |
| **Background** | 2 | Provide a background about the rationale of study, what has been previously done, and why this survey is needed. | 6 |
| **Purpose/aim** | 3 | Identify specific purposes, aims, goals, or objectives of the study. | 6 |
| **Methods** | | | |
| **Study design** | 4 | Specify the study design in the methods section with a commonly used term (e.g., cross-sectional or longitudinal). | 6 |
| | 5a | Describe the questionnaire (e.g., number of sections, number of questions, number and names of instruments used). | 6 and eMethods 3-5 |
| **Data collection methods** | 5b | Describe all questionnaire instruments that were used in the survey to measure particular concepts. Report target population, reported validity and reliability information, scoring/classification procedure, and reference links (if any). | 6-7 and eMethods 3-6 |
| | 5c | Provide information on pretesting of the questionnaire, if performed (in the article or in an online supplement). Report the method of pretesting, number of times questionnaire was pre-tested, number and demographics of participants used for pretesting, and the level of similarity of demographics between pre-testing participants and sample population. | 6 |
| | 5d | Questionnaire if possible, should be fully provided (in the article, or as appendices or as an online supplement). | eMethods 3-5 |
| **Sample characteristics** | 6a | Describe the study population (i.e., background, locations, eligibility criteria for participant inclusion in survey, exclusion criteria). | 6 and eMethods 2 |
| | 6b | Describe the sampling techniques used (e.g., single stage or multistage sampling, simple random sampling, stratified sampling, cluster sampling, convenience sampling). Specify the locations of sample participants whenever clustered sampling was applied. | 6 |
| | 6c | Provide information on sample size, along with details of sample size calculation. | 7 |
| | 6d | Describe how representative the sample is of the study population (or target population if possible), particularly for population-based surveys. | -- |
| **Survey administration** | 7a | Provide information on modes of questionnaire administration, including the type and number of contacts, the location where the survey was conducted (e.g., outpatient room or by use of online tools, such as SurveyMonkey). | 6 |
| | 7b | Provide information of survey's time frame, such as periods of recruitment, exposure, and follow-up days. | 6 |
| | 7c | Provide information on the entry process: | eMethods 7 |

© 2024 Hantel A et al. *JAMA Network Open.*

| Section/topic | Item | Item description | Reported on page # |
|---|---|---|---|
| | | –>For non-web-based surveys, provide approaches to minimize human error in data entry.<br>–>For web-based surveys, provide approaches to prevent “multiple participation” of participants. | |
| **Study preparation** | 8 | Describe any preparation process before conducting the survey (e.g., interviewers’ training process, advertising the survey). | N/A |
| **Ethical considerations** | 9a | Provide information on ethical approval for the survey if obtained, including informed consent, institutional review board [IRB] approval, Helsinki declaration, and good clinical practice [GCP] declaration (as appropriate). | 7 |
| | 9b | Provide information about survey anonymity and confidentiality and describe what mechanisms were used to protect unauthorized access. | eMethods 7 |
| **Statistical analysis** | 10a | Describe statistical methods and analytical approach. Report the statistical software that was used for data analysis. | 7 |
| | 10b | Report any modification of variables used in the analysis, along with reference (if available). | 7 and eMethods 5-6 |
| | 10c | Report details about how missing data was handled. Include rate of missing items, missing data mechanism (i.e., missing completely at random [MCAR], missing at random [MAR] or missing not at random [MNAR]) and methods used to deal with missing data (e.g., multiple imputation). | 7 |
| | 10d | State how non-response error was addressed. | 7 |
| | 10e | For longitudinal surveys, state how loss to follow-up was addressed. | N/A |
| | 10f | Indicate whether any methods such as weighting of items or propensity scores have been used to adjust for non-representativeness of the sample. | N/A |
| | 10g | Describe any sensitivity analysis conducted. | N/A |
| **Results** | | | |
| **Respondent characteristics** | 11a | Report numbers of individuals at each stage of the study. Consider using a flow diagram, if possible. | N/A |
| | 11b | Provide reasons for non-participation at each stage, if possible. | N/A |
| | 11c | Report response rate, present the definition of response rate or the formula used to calculate response rate. | 7 |
| | 11d | Provide information to define how unique visitors are determined. Report number of unique visitors along with relevant proportions (e.g., view proportion, participation proportion, completion proportion). | N/A |
| **Descriptive results** | 12 | Provide characteristics of study participants, as well as information on potential confounders and assessed outcomes. | 7, 15 |
| **Main findings** | 13a | Give unadjusted estimates and, if applicable, confounder-adjusted estimates along with 95% confidence intervals and p-values. | 7-8 |
| | 13b | For multivariable analysis, provide information on the model building process, model fit statistics, and model assumptions (as appropriate). | 8-9 and eMethods 10 |
| | 13c | Provide details about any sensitivity analysis performed. If there are considerable amount of missing data, report sensitivity analyses comparing the results of complete cases with that of the imputed dataset (if possible). | N/A |
| **Discussion** | | | |

© 2024 Hantel A et al. *JAMA Network Open.*

| Section/topic | Item | Item description | Reported on page # |
|---|---|---|---|
| **Limitations** | 14 | Discuss the limitations of the study, considering sources of potential biases and imprecisions, such as non-representativeness of sample, study design, important uncontrolled confounders. | 10 |
| **Interpretations** | 15 | Give a cautious overall interpretation of results, based on potential biases and imprecisions and suggest areas for future research. | 9-11 |
| **Generalizability** | 16 | Discuss the external validity of the results. | -- |
| **Other sections** | | | |
| **Role of funding source** | 17 | State whether any funding organization has had any roles in the survey's design, implementation, and analysis. | 12 |
| **Conflict of interest** | 18 | Declare any potential conflict of interest. | 12-13 |
| **Acknowledgements** | 19 | Provide names of organizations/persons that are acknowledged along with their contribution to the research. | 12 |

© 2024 Hantel A et al. *JAMA Network Open.*

**eTable 1.** Respondent Familiarity and Ability to Explain AI Model Types (N=203)

| Model Type | Familiar N (%) | Not familiar N (%) | Can Explain N (%) | Can't Explain N (%) |
|---|---|---|---|---|
| Pathology | 74 (36.5) | 129 (63.5) | 48 (23.6) | 155 (76.4) |
| Radiology | 112 (55.2) | 91 (44.8) | 83 (40.9) | 120 (59.1) |
| Prognostic | 108 (53.2) | 95 (46.8) | 96 (47.3) | 107 (52.7) |
| Clinical Decision | 92 (45.3) | 111 (54.7) | 80 (39.6*) | 122 (60.4*) |

*N=202

© 2024 Hantel A et al. *JAMA Network Open.*

**eTable 2.** Respondent General Predictions Related to AI (N=203)

| Prediction | Decrease N (%) | Not Change N (%) | Increase N (%) |
|---|---|---|---|
| Change in Patient Trust of Oncologists | 20 (9.9) | 141 (69.5) | 42 (20.7) |
| Change in Oncologists' Skills | 37 (18.2) | 61 (30.0) | 105 (51.7) |
| Change in Cancer Care Disparities | 43 (21.2) | 84 (41.4) | 76 (37.4) |

© 2024 Hantel A et al. *JAMA Network Open.*

**eTable 3.** Respondent Clinical Predictions Related to AI (N=203)

| Clinical Prediction | Agree<br>N (%) | Disagree<br>N (%) |
|---|---|---|
| Improve Cancer Diagnosis | 194 (95.6) | 9 (4.4) |
| Improve Cancer Treatment | 181 (89.2) | 22 (10.8) |
| Improve Side Effect Management | 122 (60.4*) | 80 (39.6*) |
| Improve End-of-Life Decision Making | 77 (38.1*) | 125 (61.9*) |

*N=202

© 2024 Hantel A et al. *JAMA Network Open.*

**eTable 4.** Respondent Views on Acceptability of Direct-to-Patient AI Model Applications (N=204)

| Model Application | Acceptable<br>N (%) | Not Acceptable<br>N (%) |
|---|---|---|
| Professional to Lay Language Translation | 107 (52.5) | 97 (47.5) |
| Clinical Trial Matching | 103 (50.5) | 101 (49.5) |
| Supportive Care Counseling | 166 (81.4) | 38 (18.6) |
| Medical Decision Aides | 113 (55.4) | 91 (44.6) |

© 2024 Hantel A et al. *JAMA Network Open.*

**eTable 5.** Bivariate Logistic Regressions Between Demographic Characteristics and Preference for Patient Consent to the Use of a Treatment Decision AI Model

| | | | | 95% Confidence Interval | |
|---|---|---|---|---|---|
| **Characteristic** | **Category** | **Odds ratio** | **p-value** | **Lower** | **Upper** |
| Age Group (N=202) | <40 years old | ref. | -- | -- | -- |
| | 40-59 years old | 0.90 | 0.82 | 0.37 | 2.20 |
| | 60-80 years old | 1.48 | 0.50 | 0.47 | 4.68 |
| | >80 years old | 1.00 | -- | -- | -- |
| Gender (N=200) | Female | ref. | -- | -- | -- |
| | Male | 0.46 | 0.07 | 0.20 | 1.07 |
| Years in Practice (N=202) | 0-5 | ref. | -- | -- | -- |
| | 6-10 | 1.50 | 0.56 | 0.38 | 5.92 |
| | 11-20 | 0.67 | 0.44 | 0.24 | 1.87 |
| | 21-30 | 2.81 | 0.17 | 0.65 | 12.26 |
| | 31+ | 0.89 | 0.86 | 0.24 | 3.33 |
| Practice Setting (N=200) | Primary Academic | ref. | -- | -- | -- |
| | Other Setting | 2.39 | 0.02 | 1.13 | 5.06 |
| Oncology Specialty (N=202) | Medical Oncology | ref. | -- | -- | -- |
| | Radiation Oncology | 1.14 | 0.85 | 0.31 | 4.22 |
| | Surgical Oncology | 0.52 | 0.11 | 0.24 | 1.14 |
| Familiar with 2+ AI Model Types (N=201) | Yes | ref. | -- | -- | -- |
| | No | 0.40 | 0.06 | 0.16 | 1.02 |
| Prior AI Training (N=202) | Yes | ref. | -- | -- | -- |
| | No | 2.81 | 0.008 | 1.32 | 6.00 |

© 2024 Hantel A et al. *JAMA Network Open.*

**eReference.**

1. Sharma A, Minh Duc NT, Luu Lam Thang T, et al. A Consensus-Based Checklist for Reporting of Survey Studies (CROSS). *Journal of General Internal Medicine*. 2021;36(10):3179-3187. doi:10.1007/s11606-021-06737-1
