# Supplementary material for: Perspectives of Oncologists on the Ethical Implications of Using Artificial Intelligence for Cancer Care
Source: JAMA Netw Open. 2024 Mar 28;7(3):e244077. doi: 10.1001/jamanetworkopen.2024.4077 (PMC10979310; doi:10.1001/jamanetworkopen.2024.4077)
Supplement: Supplement 2. — Data Sharing Statement [file jamanetwopen-e244077-s002.pdf]

Data Sharing Statement

Hantel. Perspectives of Oncologists on the Ethical Implications of Using Artificial Intelligence for Cancer Care. *JAMA Netw Open*. Published March 28, 2024. doi:10.1001/jamanetworkopen.2024.4077

Data
Data available: No

Additional Information
Explanation for why data not available: The institutional review board approval for this study does not allow for sharing of individual respondent data. A data dictionary is provided in the article's supplemental data file.
